# Association Georgia Central Railroad Surgeons

**Published:** 1894-01

**Authors:** 


### ASSOCIATION GEORGIA CENTRAL RAILROAD SURGEONS.

The Fourth Annual meeting of the Association of Georgia Central Railroad Surgeons will convene in Atlanta, Ga., Tuesday, April 17th, one day preceeding the meeting of the Medical Association of Georgia. The meeting of our Association, both in Columbus and Savannah were so successful that the Committee on Programme desire to make the Atlanta meeting as successful, if not more so, than the two preceeding meetings. In order to accomplish this, we call your attention *now* to the time and place of meeting, and ask you to aid us

Please arrange your business so that you can be with us, and contrihute to the interest of the Association by preparing a paper for the occosion on some Surgical subject of peculiar interest to railroad work. You will greatly aid the committee by sending the title of your paper at an early date to the chairman of the committee, so that the programme may be arranged·

*Titles of papers must be sent in before the* 10*th of March.*

Sincerely yours,

HOWARD J. WILLIAMS, Chairman, Macon. Ga.
ROSS P. COX, Rome, Ga.
WILLIAM S. HAYS, Allendale, S. C.
Committee on Programme.

